# Blocking human fear memory with the matrix metalloproteinase inhibitor doxycycline

**DOI:** 10.1038/mp.2017.65

**Published:** 2017-04-04

**Authors:** D R Bach, A Tzovara, J Vunder

**Affiliations:** 1 Division of Clinical Psychiatry Research, Psychiatric Hospital, University of Zurich, Zurich, Switzerland; 2 Neuroscience Centre Zurich, University of Zurich, Zurich, Switzerland; 3 Wellcome Trust Centre for Neuroimaging and Max Planck UCL Centre for Computational Psychiatry and Ageing Research, University College London, London, UK

## Abstract

Learning to predict threat is a fundamental ability of many biological organisms, and a laboratory model for anxiety disorders. Interfering with such memories in humans would be of high clinical relevance. On the basis of studies in cell cultures and slice preparations, it is hypothesised that synaptic remodelling required for threat learning involves the extracellular enzyme matrix metalloproteinase (MMP) 9. However, *in vivo* evidence for this proposal is lacking. Here we investigate human Pavlovian fear conditioning under the blood–brain barrier crossing MMP inhibitor doxycyline in a pre-registered, randomised, double-blind, placebo-controlled trial. We find that recall of threat memory, measured with fear-potentiated startle 7 days after acquisition, is attenuated by ~60% in individuals who were under doxycycline during acquisition. This threat memory impairment is also reflected in increased behavioural surprise signals to the conditioned stimulus during subsequent re-learning, and already late during initial acquisition. Our findings support an emerging view that extracellular signalling pathways are crucially required for threat memory formation. Furthermore, they suggest novel pharmacological methods for primary prevention and treatment of posttraumatic stress disorder.

## Introduction

Learning to predict threat is a fundamental ability of many biological organisms, yet in anxiety disorders dysfunctional overprediction of threat causes tremendous suffering. A dedicated threat memory system in many mammals including humans can be probed using Pavlovian discriminative fear conditioning.^1^ In this paradigm, an initially neutral cue (conditioned stimulus, CS+) is contingently paired with an aversive event (unconditioned stimulus, US), while a different cue (CS−) is not. Crucial pivot in learning US predictions is a synaptic reconfiguration that leads to long-term potentiation (LTP) of amygdala neurons with converging CS and US input.^1^ Interfering with threat memory in Pavlovian fear conditioning is being investigated as preclinical model for treatment of posttraumatic stress disorder.^2^ However, pharmacological manipulation of threat memories in humans has been difficult. The most direct mechanism of action used in non-human research—broad spectrum protein synthesis inhibition^3^—is not applicable in humans.^4^ A more specific option is propranolol, which interferes with human threat learning possibly by inhibiting synthesis of proteins required for synaptic plasticity.^2,5^ However, propranolol may be less effective in individuals with high trait anxiety.^6^ In this paper, we sought to inhibit synaptic remodelling by targeting an extracellular signalling pathway.

In the past decades, evidence has accumulated for a role of extracellular matrix in memory formation.^7,8^ In terms of its structure, extracellular matrix is organised in perineuronal nets.^9^ Their integrity is crucial for memory storage, including threat memory.^10,11,12^ Functionally, the signalling pathway that induces LTP appears to involve extracellular enzymes, and specifically matrix metalloproteinase 9 (MMP-9).^7^ In slices, MMP-9 inhibition or knockout reduces long-term potentiation.^13,14,15,16^ Interestingly, active MMP-9 alone is sufficient to induce LTP.^13,14^ *In vivo*, MMP-9 inhibition appears to impact on spatial/contextual memory in non-human mammals.^17,18^ While the precise mechanism by which MMP-9 takes part in synaptic circuit remodelling remains elusive,^7^ these findings suggest that MMP-9 may be required for formation of human fear memories. Here, we sought to impair human Pavlovian fear conditioning with the tetracycline antibiotic doxycycline, a blood–brain barrier crossing^19^ *in vivo* and *in vitro* MMP-9 inhibitor.^20,21^ To isolate effects of the drug on fear acquisition/consolidation from direct effects on retrieval, we trained participants under doxycycline or placebo, and tested fear retention 7 days later.

Unlike in rodent species, fear conditioning in humans does not elicit overt behavioural responses to the CS. It is usually quantified using readouts from the automomic nervous system such as skin conductance responses (SCR),^22,23,24^ or by its interaction with an innate startle response, termed fear-potentiated startle.^25,26,27^ The latter method is the most sensitive way to measure fear retention after initial learning,^28^ but during initial learning the presentation of startle stimuli inhibits fear acquisition.^29^ Hence our primary outcome measure was fear-potentiated startle eye blink response (SEBR) during fear retention. To quantify the progress of fear learning and re-learning where no startle stimuli were delivered, we relied on SCR. While fear-potentiated startle scales with CS/US association strength,^30^ SCR are suggested to reflect CS-specific associability,^31,32^ which is based on a weighted average of surprise about the previous outcomes following this CS.^33^

## Materials and methods

### Participants

**Figure 1:**
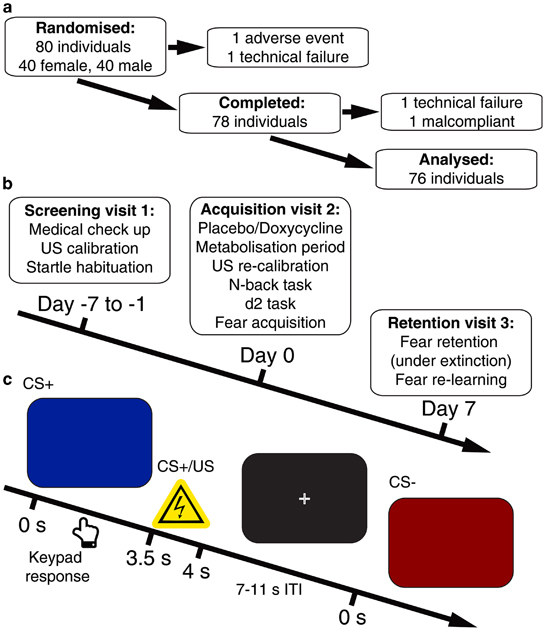
Experimental protocol. (**a**) Recruitment and exclusion of participants. (**b**) Study visit timeline. (**c**) Intra-trial procedure. A CS (red or blue screen) was presented for 4 s; 50% of CS+ co-terminated with a 0.5 s US (painful electric stimulation). CS, conditioned stimulus; US, unconditioned stimulus.

Participants were recruited from the general population (*n*=80; 40 per group; 20 female per group). Two participants did not complete acquisition visit 2: one due to vomiting immediately after ingesting the drug, and another due to an irresolvable computer failure. Two further participants were excluded from analysis: one due to failure of the startle sound equipment on visit 3, and the other did not comply with instructions and pressed response keys on <2% of trials on both visits. Re-including these participants into the analysis did not change any of the statistical inference results. The reported final sample therefore comprised 76 individuals, 38 per group (Figure 1a). The groups did not differ in age, gender, body mass index or baseline personality measures (Table 1). All participants were screened for health conditions by a physician (see Supplementary Information for in- and exclusion criteria). The study was conducted in accord with the Declaration of Helsinki and approved by the governmental research ethics committee (Kantonale Ethikkomission Zurich, KEK-ZH 2014-0669) and the Swiss Agency for Therapeutic Products (Swissmedic, Bern, Switzerland; 2015DR1136). All participants gave written informed consent using a form approved by the ethics committee. The study was pre-registered at the primary ISRCTN registry (ISRCTN66987216) and at the Swiss Federal Complementary Database (KOFAM; SNCTP000001439).

**Table 1:** Group characteristics

	*Placebo group*		*Doxycycline group*		
	*20 Male*	*18 Female*	*20 Male*	*18 Female*	
*Sex*	*Mean*	*s.d.*	*Mean*	*s.d.*	P*-Value*
Age	23.05	2.65	23.76	4.25	0.385
BMI	22.05	2.45	22.65	2.47	0.300
STAI X1	32.38	6.70	33.74	7.07	0.395
STAI X2	38.03	6.48	38.48	6.28	0.765
BDI	3.37	2.98	4.50	4.27	0.185
Current	3.55	1.21	3.72	1.27	0.552
Pain difference	−15.63	15.23	−8.35	17.70	0.101
Accuracy	0.85	0.08	0.82	0.10	0.200
Performance	0.97	0.08	0.95	0.17	0.511
d2 performance of attention	178.71	30.65	176.68	35.67	0.791
d2 speed	204.21	33.53	202.84	35.83	0.864
d2 errors	12.01	10.31	12.30	12.88	0.916

Abbreviations: BDI, Beck Depression Inventory; BMI, body mass index; Current, electric current used for the US; STAI, State-Trait Anxiety Inventory (X1: trait anxiety, X2: state anxiety); US, unconditioned stimulus. Pain difference: difference in average pain ratings of 14 stimuli before and after the acquisition test. Accuracy: % correct responses in incidental task. Performance: % responses in incidental task. d2-measures: performance of attention; speed; and % errors. p: *P*-value of a two-sample *t*-test between the two groups. Questionnaires (STAI, BDI) were filled in before drug ingestion. STAI, BDI and pain difference, were entered into the statistical model for the outcome measures as covariates.

### Power analysis

To determine required sample size, we conducted a power analysis (using G*power^34^) based on a pilot study with the same setup,^28^ in which the effect size for a CS+/CS− SEBR difference in an untreated control group was (Cohen's) d=1.17. Under the assumption of equal variance in a doxycycline-treated group, a fear memory reduction of 50% would correspond to an effect size of d=0.59. Thus, a sample size of *N*=74 was required to achieve 80% power at an alpha rate of 0.05. We recruited *N*=80 participants to allow for attrition.

### Study medication

The study medication was doxycycline, brand name Vibramycin (Pfizer, Zurich, Switzerland). The study dose of 200 mg was based on the smallest antibiotic dose recommended by the manufacturer, in order to reduce side effects. Peak cerebrospinal fluid concentrations are reached at approximately 180 min after oral administration.^19^ The drug's half-life is ~16 h according to manufacturer's information; such the drug was cleared by more than 99.9% at the retention test 7 days after ingestion. A GMP-licensed pharmacy (Kantonsapotheke, Zurich, Switzerland) manufactured, blinded and randomised the study medication separately for males and females; mannitol was used as placebo. Randomisation code was broken after the last participant completed the study, and after all data were checked for consistency.

### Procedure

*Screening visit 1 (day −7 to day −1)*

Study procedure is summarised in Figure 1b. On visit 1, we determined US intensity and habituated participants to startle sounds, performed medical examination to check exclusion criteria (Supplementary Information), and measured weight/height to compute body mass index.

*Acquisition visit 2 (day 0)*

Acquisition visit 2 took part in the morning hours between 0800 and 1300 hours. Participants filled in the German translations of the State-Trait Anxiety Inventory^35^ and Beck's Depression Inventory^36^ before ingesting the study medication. During a 180-min metabolisation interval, they were kept under surveillance of study staff. Next, participants performed a 15-minute N-back working memory task and a paper-and-pencil version of the d2 attention test. Then the fear acquisition protocol started. This was a standard discriminant delay conditioning paradigm with 160 trials (80 CS+, 80 CS−) in two blocks (Figure 1c). The CS+ co-terminated with an electric stimulation as aversive US (Supplementary Information) in 50% of trials. CS were a blue or red screen background presented for 4 s, while the screen was black during the inter trial interval, randomly determined to be 7, 9 or 11 s. Trial sequence was randomly balanced for each participant, with the restriction that the first trial of each block was always a reinforced CS+. As an incidental task, participants were instructed to press a key with right index or middle finger to indicate CS colour. Colour-CS and colour-button associations were balanced across participants. Colour-CS association had no impact on any outcome measure.

*Retention visit 3 (day 7)*

Participants were instructed that they might receive US, but that CS/US contingency was determined by the computer and unknown to the study assistant. They saw 40 CS (20 CS+/20 CS−) in randomly balanced order, and heard a startle probe (Supplementary Information) 3.5 s after onset of all CS, but never received a US. Note that the motoric startle response makes SCR data from this session unusable. Immediately afterwards, we measured re-learning over 80 trials by co-terminating 50% of CS with a US over 80 trials, but without startle sounds.

*N-back task*

Random letters were shown on the screen for 500 ms followed by a fixation cross for 2500 ms. Participants were tasked to indicate on each trial whether the letter matched the one from N steps back. N was constant (1/2/3) within each of three blocks.^37^ Each block contained 70 non-targets and 30 targets.

*d2 test*

The d2-test^38^ is designed to measure sustained attention over 5 min. Participants are given 20 seconds to work on each of 14 rows; in each row their task is to cross every letter 'd' marked with two lines above and/or below, while leaving out the letter 'd' not marked with two lines, as well as the letter 'p'.

*Outcome measures*

Primary outcome measure was startle potentiation during the retention test, measured as SEBR from orbicularis oculi electromyogram (Supplementary Information). There were no missing data in the primary outcome. Secondary outcome measure was SCR. No SCR data were available for three participants during acquisition (one placebo, two doxycycline) and for one participant during re-learning (placebo), due to electrode detachment. Because of artefacts, a small number of individual trials in some participants were excluded (<1.5% of trials, see Supplementary Information for details).

### Psychophysiological modelling

For psychophysiological analysis, we used a Matlab toolbox for psychophysiological modelling, PsPM 3.0 (pspm.sourceforge.net). SEBR processing was performed using the most sensitive method from a previous methodological comparison using the same setup^28^ (Supplementary Information). This procedure builds on a psychophysiological model (PsPM)^39^ and quantifies, for each trial, amplitude of the SEBR by linear regression onto a canonical SEBR with variable onset.^28^ Skin conductance was analysed by nonlinear inversion of a PsPM that describes the anticipatory SCR^23,24^ under a canonical response function.^40,41^

### Statistical analysis

Statistical analysis was done in R (www.r-project.org), version 3.3.1, using aov() for analysis of variance (ANOVA). R package nlme, version 3.1.128, was used for linear mixed effects (LME) models (Supplementary Information).^42^ We analysed trial-wise SEBR in a 2 (drug) × 2 (CS+/CS−) × 20 (trial) multistratum repeated-measures ANOVA. Because trial sequence was not the same for each participant, habituation may affect CS+ and CS− differently in the two groups. Therefore, results were confirmed in a LME model that accounts for the linear effect of time (trial number across CS). For SCR, only trials without US entered analysis. As there are uneven numbers of CS+US- and CS− trials, we averaged within mini-blocks of 10 trials, and entered these averages into a 2 (drug) × 2 (CS+/CS−) × 16 (mini-block) repeated-measures ANOVA (acquisition) or 2 (drug) × 2 (CS+/CS−) × 8 ANOVA (re-learning). Participants for whom all trials in at least one mini-block were missing were excluded (Supplementary Information). Results were confirmed in a trial-wise LME, in which missing data points were removed on a trial-by-trial basis, as this model can deal with unbalanced data.

*N-back task*

Performance was averaged within conditions and analysed in a 3 (N-back) x 2 (target/non-target) repeated-measures ANOVA.

*Control measures*

Control measures were tested for group differences with independent samples *t*-tests, without correction for multiple comparison. We tested the following measures: age, weight, body mass index, Beck’s Depression Inventory sum score, STAI sum scores, electric current used as US, difference in averaged ratings of the same 14 pain stimuli before and after the acquisition test. Further, we tested the following three outcomes of the d2-test: performance of attention (marked—missed targets), speed (total number of targets processed), error percentage (all errors divided by total processed targets). During fear acquisition, we analysed performance (any key pressed), accuracy (correct key pressed) and reaction times. No *t*-test for group differences on any control measure yielded a statistically significant result. Entering anxiety and depression scores, or pain habituation during the acquisition session, into the statistical model as a covariate, together with the covariate × CS interaction, had no impact on statistical significance of the primary outcome (interaction drug × CS during retention).

## Results

**Figure 2:**
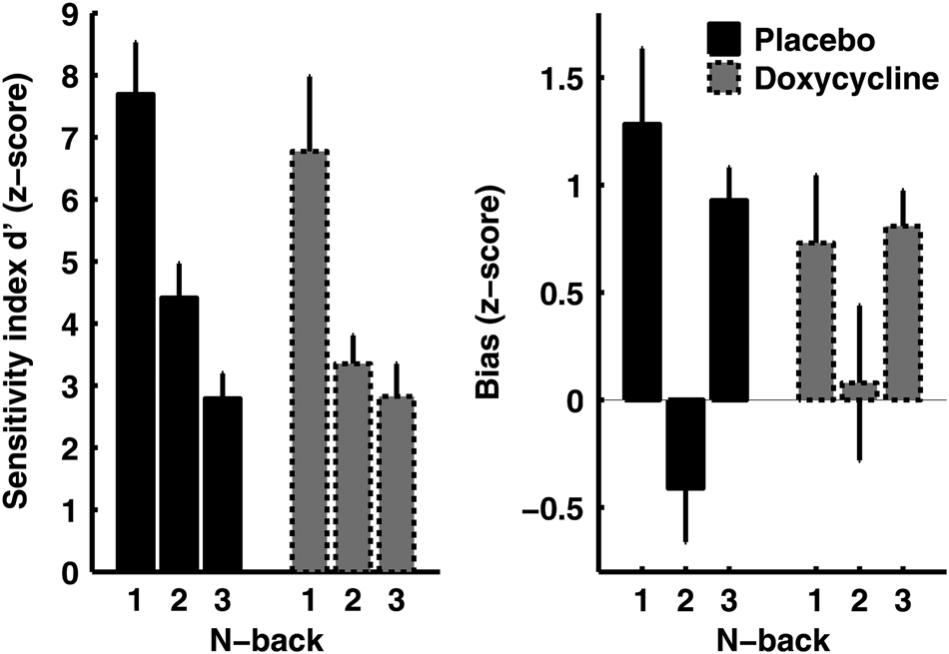
N-back task accuracy, transformed to Sensitivity index d'=z(Hit Rate)−z(False Alarm Rate), and Bias=1/2*(z(Hit Rate)+z(False Alarm Rate)). Targets for this transformation are letter repetitions, i.e., a positive bias implies that participants were more likely to indicate 'same letter' than 'different letter'. There was no statistically significant impact of doxycycline on d' or bias (Supplementary Information).

Three-and-a-half hours after orally ingesting placebo or 200 mg doxycyline, participants performed a discriminant delay fear conditioning task (Figure 1c) in which the CS+ co-terminated with an aversive electrical stimulation in 50% of trials, while the CS− was never reinforced. To reduce a potential impact of variability in learning speed, we overtrained participants in 160 trials (80 CS+, 80 CS−). Before the fear acquisition task started, we ensured that doxycycline had no impact on pain perception, attention in the d2 test, or memory on a seconds timescale in a 1/2/3-back task (see Materials and Methods, Table 1, Figure 2 and Supplementary Information). Performance in an incidental task during fear acquisition was also unchanged by the drug.

**Figure 3:**
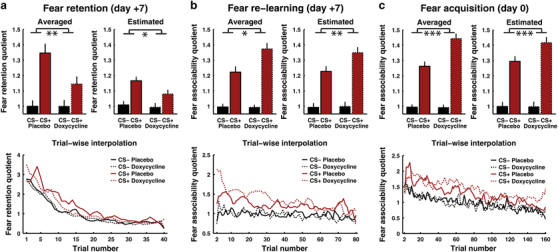
Fear retention under extinction, and fear associability during re-learning and initial acquisition. (**a**) Fear retention quotient, based on measured SEBR in a fear retention session 7 days after acquisition. Condition averages with standard errors from *N*=76 participants and 40 trials per participant; estimated marginal means and standard errors from LME model; and trial-wise interpolated and averaged data. (**b**) Fear associability quotient from fear re-learning immediately after the retention test, based on measured SCR. Condition averages with standard errors from *N*=71 participants and eight mini-blocks per participant; estimated marginal means and standard errors from a trial-by-trial LME model, on 4433 trials from *N*=75 participants; and trial-wise interpolated and averaged data (no data available for trial 1 which was always reinforced). (**c**) Fear associability quotient from acquisition session: *N*=65 participants and 16 mini-blocks per participant; 8646 trials from *N*=73 participants. Full statistical results can be found in Supplementary Information. LME, linear mixed effect; SCR, skin conductance responses; SEBR, startle eye blink response. *P<0.05; **P<0.01; ***P<0.001.

Primary outcome was fear retention under extinction, 7 days after acquisition (see Figure 3a and Supplementary Information). Fear-potentiated startle was measured as SEBR to acoustic startle probes on each of 40 extinction trials, and analysed in a repeated-measures ANOVA. Threat memory (that is, CS+/CS− difference in SEBR) was attenuated by ~60% in the doxycycline as compared to placebo group (interaction drug × CS: *P*=0.01). This initial analysis did not take into account that the sequence of CS+ and CS− was randomised for each participant, and might have been slightly different between the two groups. This is why we replicated these results in a LME model with trial number as predictor across CS types (see Supplementary Information for details). This analysis revealed the same group difference in threat memory (interaction drug × CS: *P*=0.021). Finally, there was no difference between the groups in terms of extinction (that is, no drug × trial × CS interaction).

Next, we analysed the re-learning session, which immediately followed the retention session and always started with a reinforced CS+ trial. No startle probes were used during this session in order to allow unimpaired learning.^29^ Instead we analysed SCR, which are thought to reflect a Pearce-Hall type associability signal,^33^ that is, a running average of surprise about the outcome on previous trials.^31,32^ If fear memory were weak during the non-reinforced retention test, one would expect greater surprise about the presence of a US during the reinforced re-learning session. This would imply a larger CS+/CS− difference in SCR, particular in the beginning of the re-learning session. Indeed during re-learning, SCR surprise signals were larger in the doxycycline than in the placebo group in a block-wise ANOVA that was ignorant about trial sequence, and in a LME model that took account of trial sequence (interaction drug × CS; ANOVA: *P*=0.010; LME: *P*<0.001; Figure 3b and Supplementary Information). The more sensitive LME model furthermore indicated that enlarged surprise signals upon CS+ presentation in the doxycyline group decreased over time (interaction drug × CS × time; *P* =0.028), as expected (see Figure 3b and Supplementary Information). These findings are consistent with weaker threat memory at the start of the re-learning session.

Finally, we sought to elucidate whether doxycycline acts on memory consolidation after acquisition alone, or whether an effect of the drug was already observable during acquisition. This was an exploratory analysis. As in the re-learning session, we found a larger CS+/CS− difference in SCR in the doxycycline than placebo group, potentially indicating greater surprise about the presence of the US in this group (interaction drug × CS; ANOVA: *P*<0.001; LME: *P*<0.001; see Figure 3c and Supplementary Information). This pattern could be explained if the neural system, for each of the CS, keeps track of recent surprise about CS outcome, but fails to appropriately update outcome predictions. Such model is in keeping with our initial result that performance in a 1/2/3-back task, addressing memory on a timescale of seconds, was intact in the doxycycline group. According to this interpretation, the group difference in surprise signals should be small in the beginning of the session (when both groups have not yet formed CS/US associations) and increase towards the end (when the placebo group has formed stronger associations). This was indeed the case: The LME results indicated that enlarged surprise signals upon CS+ presentation in the doxycyline group were not found in earlier but in later trials of the learning session (interaction Drug × CS × Time; *P*=0.021), and this appeared to start after about 40 trials (see Figure 3c and Supplementary Information).

## Discussion

In this paper, we addressed inhibition of human fear conditioning with the MMP inhibitor doxcycyline. In the primary outcome, fear-potentiated startle, we found reduced fear memory retention in those participants who were trained under doxycycline. This attenuated fear memory was also evident by increased surprise to the CS+ during re-learning directly after the retention test, as compared to a placebo group. Because doxycycline was ingested before the acquisition session, this raises a question as to whether doxycycline impacts already on threat memory acquisition, or only later on consolidation. Analysis of the acquisition session provides some evidence for the former view, that is, that doxycycline impacts on threat memory already during training session. However, it does not impair sensory memory on a timescale of seconds, as shown by unimpaired performance in an N-back task.

Doxcycline is a potent inhibitor of human MMP-9 and other MMPs.^20,21^ Our results are in keeping with a model in which human amygdala-dependent threat memory requires extracellular MMP signalling for synaptic remodelling. Because in our human model we cannot conclusively rule out that other molecular targets of doxycycline contribute to memory impairment, converging evidence with other human MMP inhibitors would be desirable. Doxycycline has been reported to interact with mitochondrial function,^43^ and less consistently, with mammalian protein synthesis.^44^ However, a possible relation of these molecular targets to synaptic plasticity or to memory formation is not established. They therefore appear as less likely to underlie the results reported here. Whether doxycycline also impacts on human hippocampus-dependent, spatial or semantic memory, as previously hypothesised,^17,18^ remains to be determined. Early human work performed in the context of sleep research has provided some hints that doxycycline may have an impact on semantic memory^45^ but also pointed towards a potential interaction with rapid eye movement sleep.^46^

Our primary outcome, fear-potentiated startle, is a well-established measure of fear memory strength in many species,^25,47,48,49,50,51^ such that the impact of doxycycline on fear retention in this measure can be unambiguously interpreted. In contrast, our analysis of its effect on fear acquisition relies on SCR. Under native conditions, averaged SCR across an entire experiment are often taken as in index of association strength,^22^ but their fluctuations over time appear to be more consistent with an associability signal.^31,32^ The former view is justified because on average, associability is higher for a partially reinforced CS+ than a CS−, but under a pharmacological manipulation, associability and association strength may be decoupled. In the current experiment, it appears that sensory memory is intact under doxycyline, as indexed by results from the 1/2/3-back task. We suggest that the fear learning system stores a running average of surprise about previous trial outcomes, but that it fails to appropriately update threat predictions. This would lead to a continued occurrence of unpredicted US, which would be reflected in higher associability of CS+ in the doxcycline group. In the placebo group, after establishing the threat prediction, SCR to the CS+ would tend to habituate faster as the US becomes predicted and associability decreases. Indeed, we show that SCR to CS+ are greater in the doxycycline than placebo group after about 40 trials into the experiment. Our interpretation relies on an impact of doxycline on updating threat predictions, but not storage of previous trial outcomes. This is in keeping with an impact of MMP-9 inhibitors on late LTP, but not on other synaptic plasticity mechanisms such as early LTP and paired-pulse facilitation,^7^ but requires confirmation on a molecular level.

While evidence for a role of MMP-9 in LTP is mounting, its mechanism of action is unknown. In slices and cell cultures, MMP-9 appears to be transported to synapses at times of neural activity,^52^ colocalises with NMDA- and AMPA-receptors,^53^ and impacts on spine re-modelling.^14^ However, its proteolytic target remains elusive. It has been hypothesised that MMP-9 activates a signalling pathway that ultimately leads integrins to direct AMPA receptors into the synaptic membrane.^7^ Others have proposed that MMP-9 is involved in remodelling extracellular matrix structure which according to this model enjoys a fundamental role in memory storage^8^ and enables long-term stability of threat memories, which—after hour-timescale consolidation—last up to a lifetime.^54^

Such uncertainty notwithstanding, our findings have potentially direct therapeutic implications. They suggest that tetracycline antibiotics—all of which are MMP inhibitors^20^—could be used for primary prevention of fear memory acquisition in persons known in advance to be potentially exposed to trauma. Furthermore, it is known that retrieval of fear memory, for example, by presentation of a CS+ without US—renders this memory labile.^3^ Subsequent re-consolidation is a protein synthesis-dependent process,^3^ and it has been suggested that pharmacological disruption of this process may specifically erase human fear memory.^5^ This would render the combination of specific fear re-activation and pharmacological intervention a potential treatment principle for posttraumatic stress disorder. It is not known whether re-consolidation involves the same signalling cascades as required for initial consolidation; yet there is a suggestion that broad-band MMP inhibition may interfere with fear memory re-consolidation in rodents.^55^ Hence, impairing human fear re-consolidation with MMP-9 inhibitors is a potential target for further research. This could complement recent efforts^2^ at finding novel strategies for the treatment of posttraumatic stress disorder, and other anxiety disorders.

## Electronic supplementary material

Supplementary Information

## Electronic supplementary material

**Supplementary Information** accompanies the paper on the Molecular Psychiatry website 10.1038/mp.2017.65
