# Supplementary material for: Blocking human fear memory with the matrix metalloproteinase inhibitor doxycycline
Source: Mol Psychiatry. 2017 Apr 4;23(7):1584–9. doi: 10.1038/mp.2017.65 (PMC5507298; doi:10.1038/mp.2017.65)
Supplement: Supplementary file 1 — Supplementary Information [file 41380_2018_134_MOESM1_ESM.pdf]

Supplementary Information for:

Blocking human fear memory with the matrix metalloproteinase inhibitor doxycycline

Dominik R Bach, Athina Tzovara, Johanna Vunder

Molecular Psychiatry, 2017

Supplementary Information

Materials and methods

Unconditioned stimulus: The unconditioned stimulus (US) was a 500 ms train of 250 electrical square pulses with an individual pulse duration of 0.2 ms, delivered on participants' dominant forearm through a pin-cathode/ring-anode configuration with a constant current stimulator (Digitimer DS7A, Digitimer, Welwyn Garden City, UK). The current was set such that perceived shock intensity was around 90% of the pain threshold. We initially (visit 1) estimated the pain threshold during two phases. First, the intensity was increased from being unperceivable to a painful level. This was set as upper limit for all following perception tests, in which participants were asked to rate the perceived intensity of 14 stimuli with different currents. Ratings were interpolated to estimate the current that the participant would have rated as 90%. This current was then individually adjusted to yield a clearly discomforting but not painful stimulus. A photograph of US electrode positioning was taken on screening visit 1 to ensure similar positioning on the other visits. On acquisition visit 2, US electrode was attached before N-back task, and US perception controlled with 14 stimuli of random intensity. Stimulation strength was modified if necessary to yield a clearly discomforting but not painful stimulus. On retention visit 3, no US were delivered before the tasks started. In both acquisition visit 2 and retention visit 3, pain perception was controlled after the task using 14 random stimuli.

Startle probes: In accordance with current recommendations[1] and our own previous work[2], white noise bursts (loudness: 102 dB, measured rise and fall time: < 2 ms, sampling frequency 44.1 kHz), were used as startle probes and delivered via headphones (Sennheiser HD 201, Germany), using the PC's inbuilt sound card (Realtek high definition audio) and an external sound amplifier (K4102, Velleman, Belgium). Sound volume was determined offline using a white noise sound of 2 s duration and a sound level meter (SL-200, Voltcraft, Germany). Sound onset was controlled by recording the output of the sound card together with EMG, and all analyses relate to the measured startle sound onset.

Supplementary Information for:

Blocking human fear memory with the matrix metalloproteinase inhibitor doxycycline

Dominik R Bach, Athina Tzovara, Johanna Vunder

Molecular Psychiatry, 2017

Psychophysiological recordings: SEBR were recorded using electromyogram (EMG) from the orbicularis oculi muscle of participants' right eye and two 4 mm Ag/AgCl cup electrodes filled with high-conductance gel. One of them was placed 10 mm below the lower eyelid in line with the pupil in forward gaze and the other on the external canthus, at a distance of 10 mm from the first[1]. Electromyogram was amplified with a Colbourn isolated bioamplifier (V75-11, Colbourn Instruments, Whitehall, PA, US). Skin conductance was recorded from the thenar/hypothenar of participants' left hand, using 8 mm Ag/AgCl cup electrodes (EL258, Biopac Systems Inc., Goleta, CA, US) and 0.5% NaCl gel (GEL101, Biopac)[3]. Skin conductance signal was amplified with an SCR coupler/amplifier (V71-23, Coulbourn Instruments). All data were digitised at 1000 Hz using a DI-149 A/D card (Dataq Instruments, Akron, OH, US), and recorded with Windaq (Dataq Instruments) software.

Psychophysiological data analysis: Analysis was done using the matlab toolbox for psychophysiological modelling, PsPM 3.0 (pspm.sourceforge.net). We band pass filtered the EMG signal with a 4th order Butterworth filter and filter band 50-470 Hz, and applied a notch filter to remove 50 Hz harmonics. Filtered EMG data were rectified and smoothed with a 3 ms (53.05 Hz) 4th order Butterworth low pass filter. Differences in electrode impedance and muscle anatomy will result in a multiplicative scaling of the true SEBR. In line with recommendations[1], we thus normalised data by dividing each participant's single-trial SEBR estimates through the mean SEBR in CS- trials.

SCR data were visually inspected by a rater blind to placebo/doxycycline condition, and artefact periods (temporary electrode detachment or signal clipping) were excluded. For the acquisition session, this removed (across participants) 16 trials (0.3%) in the placebo group and 98 trials (2.3%) in the doxycycline group; for the re-learning session, this removed 11 trials (0.5%) in the placebo group and 56 trials (2.5%) in the doxycycline group. For some of these participants, no data was available in at least one condition in one mini-block; these were excluded from ANOVA (acquisition: 3 in placebo and 5 in doxycycline group; re-learning: 1 in placebo and 3 in doxycycline group). SCR data were then filtered with a 1st order bidirectional band-pass Butterworth filter (cut-off frequencies: 0.0159 Hz - 5 Hz), and down-sampled to 10 Hz. Specifically, a fixed-latency response at CS onset and a fixed-latency

response at (potential) US onset were estimated for each trial. The inversion algorithm was not informed about trial type or the presence of an US. This method has been successfully used for quantifying fear memory in similar studies setups[4, 5]. We included only non-reinforced trials in the analysis to avoid any contamination by US responses. To account for interindividual differences in skin properties, trial-by-trial SCR were divided by the mean SCR in CS- trials, analogous to SEBR analysis.
For visualisation of trial-by-trial data in figure 3 of the main text, we accounted for the subject-specific trial sequence. For every participant and condition (CS-/CS+), we used the existing data and their trial indices, to linearly interpolate all other possible trial indices in that condition. Data were then averaged across participant.
LME models: All LME models included fixed effects for drug, CS, drug x CS and the linear effect of time (the trial number across CS), together with random intercept and time effect (R model formula: startle ~ drug*CS*time, random = 1+time|subject). Random CS effects were not included as data are normalised for the CS-, and therefore subject-specific intercept and subject-specific CS effect are collinear. Modelling higher polynomials of time, or removing the linear effect of time, or removing any of the random effects, decreased model fit in terms of Akaike information criterion (AIC) and Bayesian information criterion (BIC), both for SEBR and SCR. There was no evidence for non-normal distribution of residuals, or for heteroskedasticity, in either measure. Fixed effects statistics were extracted using the function anova().

Supplementary Information for:

Blocking human fear memory with the matrix metalloproteinase inhibitor doxycycline

Dominik R Bach, Athina Tzovara, Johanna Vunder

Molecular Psychiatry, 2017

Supplementary Tables

Table S1: Participant selection

Inclusion criteria
Healthy, German-speaking, between 18 and 40 years of age
Exclusion criteria
Contraindication to doxycycline or mannitol (history of allergic reactions)<br>Drug use in the two weeks prior to the study (exception: contraceptive drugs and incidental use of NSARs/paracetamol)<br>Known or suspected use of illicit drugs, use of benzodiazepines, alcohol abuse<br>Any history of psychiatric, neurological, or systemic/rheumatic disease<br>Other clinically significant concomitant disease states<br>Pregnant or breast-feeding women, intention to become pregnant, lack of safe contraception method<br>Participation in any other drug study within the 30 days preceding and during the present study
Blood parameters screened
Blood cell count, electrolytes, C-reactive protein, aspartate aminotransferase (ASAT/GOT), alanine aminotransferase (ALAT/GPT), gamma-glutamyl transferase (gamma-GT), kreatinin, thyroid-stimulating hormone (TSH), free thyroxine (FT4)
Urine parameters screened
Amphetamines, barbiturates, benzodiazepines, tetrahydrocannabinol, cocaine, methadone, opioids; women: beta human chorionic gonadotropin (beta-HCG) pregnancy test

Table S2: N-back task accuracy from a Drug x N (1/2/3-back) x Target (same/different) repeated-measures ANOVA. Supplemental ANOVA of transformed accuracy (d' and bias, figure 2 in main text) revealed no effect of drug either.

	F	df	p
**Drug**	< 1	1, 71	0.511
**N (1/2/3-back)**	45.23	2, 355	< 0.0001*
**Target (same/different)**	140.00	1, 355	< 0.0001*
**Drug x N**	< 1	2, 355	0.915
**Drug x Target**	1.05	1, 355	0.306
**N x Target**	35.20	2, 355	< 0.0001*
**Drug x N x Target**	< 1	2, 355	0.389

Table S3: Statistical results. Results from trial-wise (SEBR) or block-wise (SCR) drug x CS x time (trial/block) repeated measures ANOVAs, and from the best-fitting trial-wise drug x CS x time (trial) linear mixed effects (LME) models that contained a linear effect of trial number across both conditions. [* p < .05]

	**ANOVA**			**Linear mixed effects model**		
	F	df	p	F	df	p
**Fear retention (SEBR)**						
**Drug**	2.27	1, 74	0.1360	3.49	1, 74	0.0656
**CS**	39.27	1, 2886	<0.0001*	45.48	1, 2958	<0.0001*
**Time**	66.06	19, 2886	<0.0001*	129.33	2, 2958	<0.0001*
**Drug x CS**	6.66	1, 2886	0.0099*	5.31	1, 2958	0.0212*
**Drug x Time**	< 1	19, 2886	0.6868	< 1	2, 2958	0.8593
**CS x Time**	1.66	19, 2886	0.0363*	14.91	2, 2958	0.0001*
**Drug x CS x Time**	1.40	19, 2886	0.1153	2.22	1, 2958	0.1363
**Fear re-learning (SCR)**						
**Drug**	1.53	1, 69	0.2210	1.03	1, 73	0.3142
**CS**	98.70	1, 1035	<0.0001*	209.79	1, 4352	<0.0001*
**Time**	5.83	7, 1035	<0.0001*	11.40	1, 4352	0.0007*
**Drug x CS**	5.78	1, 1035	0.0164*	7.91	1, 4352	0.0049*
**Drug x Time**	1.23	7, 1035	0.2835	1.64	1, 4352	0.1998
**CS x Time**	2.66	7, 1035	0.0098*	25.00	1, 4352	<0.0001*
**Drug x CS x Time**	< 1	7, 1035	0.7333	4.86	1, 4352	0.0276*
**Fear acquisition (SCR)**						
**Drug**	1.98	1, 63	0.1650	1.17	1, 71	0.2831
**CS**	217.38	1, 1953	<0.0001*	484.12	1, 8567	<0.0001*
**Time**	29.21	15, 1953	<0.0001*	103.67	1, 8567	<0.0001*
**Drug x CS**	14.06	1, 1953	0.0002*	14.76	1, 8567	0.0001*
**Drug x Time**	< 1	15, 1953	0.6998	1.20	1, 8567	0.2741
**CS x Time**	1.58	15, 1953	0.0708	38.06	1, 8567	<0.0001*
**Drug x CS x Time**	1.03	15, 1953	0.4203	5.34	1, 8567	0.0209*

Supplementary References

1. Blumenthal TD, Cuthbert BN, Filion DL, Hackley S, Lipp OV, Van Boxtel A. Committee report: Guidelines for human startle eyeblink electromyographic studies. *Psychophysiology* 2005; **42**(1)**:** 1-15.

2. Khemka S, Tzovara A, Gerster S, Quednow BB, Bach DR. Modeling startle eyeblink electromyogram to assess fear learning. *Psychophysiology* 2017; **54**(2)**:** 204-214.

3. Hygge S, Hugdahl K. Skin conductance recordings and the NaCl concentration of the electrolyte. *Psychophysiology* 1985; **22**(3)**:** 365-367.

4. Bach DR, Daunizeau J, Friston KJ, Dolan RJ. Dynamic causal modelling of anticipatory skin conductance responses. *Biological psychology* 2010; **85**(1)**:** 163-170.

5. Staib M, Castegnetti G, Bach DR. Optimising a model-based approach to inferring fear learning from skin conductance responses. *J Neurosci Methods* 2015; **255:** 131-138.
